# Drug-Drug Interactions and Their Association With Quality of Life in Patients With Hypertension

**DOI:** 10.7759/cureus.56526

**Published:** 2024-03-20

**Authors:** Nermin Gürel, Yağız Üresin, Selçuk Şen

**Affiliations:** 1 Pharmacology and Therapeutics, Good Clinical Practice and Research Center, Mehmet Akif Ersoy Thoracic and Cardiovascular Surgery Training and Research Hospital, Istanbul, TUR; 2 Medical Pharmacology, Division of Clinical Pharmacology, Istanbul Faculty of Medicine, Istanbul University, Istanbul, TUR

**Keywords:** body mass index, age, quality of life, drug interaction, antihypertensive, hypertension

## Abstract

Introduction

This study aimed to evaluate drug-drug interactions (DDIs) and their association with the quality of life in patients with hypertension.

Materials and methods

This cross-sectional study included 123 patients with hypertension. DDIs were evaluated using the Medscape Drug Interaction Checker Database (Medscape, New York, NY). The EuroQol-5D (EQ-5D) Quality of Life Scale was used for each patient.

Results

The overall blood pressure control rate (systolic/diastolic blood pressure levels, <140/90 mmHg) was 43% (53/123). The age of the patients with uncontrolled hypertension was higher than the patients with controlled hypertension (63.67 ± 11.00 vs. 58.42 ± 10.07 years; p = 0.007). The number of DDIs showed significant correlations, positively with age (r = 0.303, p = 0.001), total number of drugs (r = 0.784, p < 0.001), number of antihypertensive drugs (r = 0.640, p < 0.001), and body mass index (BMI) (r = 0.321, p < 0.001) and inversely with EQ-5D index score (r = −0.247, p = 0.006). The EQ-5D index and visual analog score were inversely correlated with age and BMI. Additional significant linear correlations between age and the total number of drugs, age and number of the antihypertensive drugs, the number of antihypertensive drugs and BMI, and the number of total drugs and BMI were detected. Of a total of 511 identified DDIs, 14 interactions in 12 patients were considered serious, 402 interactions in 82 patients were considered significant, and 95 interactions in 39 patients were considered minor.

Conclusions

This study supports that DDIs have important associations with antihypertensive treatment and the quality of life of patients. Higher age and BMI values were associated with a higher risk of DDIs and lower quality of life in patients with hypertension.

## Introduction

Hypertension is the most known early-onset form of cardiovascular disease, and insufficient blood pressure control is a global public health challenge and is associated with cardiovascular mortality and morbidity [1]. In contrast to this well-known influence, the success rate of the treatment in patients with hypertension is not at a satisfactory level [2]. Therefore, enhancement of the success of antihypertensive treatment is the main target globally [3]. The treatment of hypertension, especially in patients with markedly high baseline blood pressure levels, is required to initiate combinations of antihypertensive drugs to achieve target blood pressure levels [4]. On the other hand, concomitant treatments related to the high prevalence of diabetes mellitus, dyslipidemia, and other chronic diseases are very frequently encountered in patients with hypertension [5]. There are two major concerns regarding drug-drug interactions (DDIs) potentially caused in the treatment of hypertension. First, the DDIs can cause an alteration of the therapeutic effect of antihypertensive treatment. The second is based on safety concerns that DDIs can increase the risk of unexpected adverse reactions to either or both drugs.

In the case of any condition that requires multiple drug treatments, the management of DDIs is a critical challenge faced by physicians in daily practice. In addition to the lack of awareness, the rapidly increasing scientific knowledge and newly defined potential DDIs make the follow-up and management of DDIs difficult. In this sense, the involvement of clinical pharmacology approaches in the management of chronic diseases is required in some cases. The use of DDI tools and databases has gained considerable momentum in recent years. As an updated and easy-to-use tool, the Medscape Drug Interaction Checker (Medscape, New York, NY) [6] is one of the best-known and freely publicly available databases.

In this study, our primary aim was to evaluate the DDIs and their association with quality of life in patients with hypertension. Additionally, sociodemographic parameters, the success of antihypertensive treatment, comorbidities, concomitant treatments, the association of DDIs with these variables, and the severity/mechanism of DDIs were analyzed.

## Materials and methods

Consequently, 123 patients with hypertension who presented to the Istanbul Faculty of Medicine Clinical Pharmacology Polyclinic were included in this cross-sectional study. The study was approved by the Istanbul Faculty of Medicine Clinical Research Ethics Committee (approval no: 1408), and all enrolled patients gave written informed consent. The study was conducted according to the Declaration of Helsinki and Good Clinical Practices rules. Sociodemographic information, blood pressure levels, body mass index (BMI) values, antihypertensive medications, comorbid conditions, concomitant drugs, and the quality of life of the patients were evaluated. The study participants were stratified into controlled and uncontrolled hypertension groups using a cut-off point of systolic blood pressure (SBP)/diastolic blood pressure (DBP) levels ≥140/90 mmHg. Those collected parameters and the number of DDIs were compared between controlled and uncontrolled patients. Additionally, the correlations between the study parameters were evaluated.

Measurement of blood pressure

Blood pressure levels were measured using a clinical-trial-use approved and calibrated blood pressure measurement device after resting for a minimum of five minutes in the sitting position. The patients were not allowed to take caffeine-containing drinks or smoke cigarettes 30 minutes before the measurement. Two measurements were performed at five-minute intervals, and an average of the two measurements was recorded. Patients with SBP and DBP levels ≥140/90 mmHg were considered as having uncontrolled hypertension.

Evaluation of drug-drug interactions

DDIs were evaluated using the Medscape Drug Interaction Checker [6]. First, the presence or absence of any interaction was screened. Then, DDIs were classified according to the severity of interactions as minor, significant (monitor closely), serious (use an alternative medication), and contraindicated. The mechanism of interaction and recommendation were overviewed in detail. 

Evaluation of quality of life

The EuroQol-5D (EQ-5D) Quality of Life Scale [7], consisting of five questions, and a visual analog scale (VAS) was used. The EQ-5D index score and VAS score were evaluated separately. The EQ-5D index score was calculated using the answers given by the patients to the three-option (no problems, moderate problems, and extreme problems) questions, and the “index calculator” was used as recommended in the guideline of the scale. For the EQ-VAS score, the patients were requested to mark a value on the ruler-like scale after being informed that “0” indicated the worst possible health level and “100” was the best possible health level. Before starting the study, permission to use the scale was obtained from the EuroQol Group Foundation.

Statistical analysis

Statistical analyses were conducted using IBM SPSS Statistics, version 21.0 (IBM Corp., Armonk, NY). The Shapiro-Wilk test was used for evaluating the distribution of variables. T-test and Pearson’s correlation analyses were used for variables with normal distribution. The Mann-Whitney U test and Spearman’s rho correlation analysis were used for variables without normal distribution. The results are presented as mean ± standard deviation (SD) and median (25th-75th percentiles). The p-values of <0.05 were considered statistically significant.

## Results

A total of 123 patients with hypertension (82 females and 41 males), with a mean age of 61.41 ± 10.89 years, participated in this cross-sectional study. Among the study participants, the blood pressure control rate (SBP/DBP, <140/90 mmHg) was 43% (53/123). The most frequent concomitant conditions were dyslipidemia (50/123) and diabetes mellitus (45/123).

The differences between patients having controlled and uncontrolled blood pressure

In addition to significantly higher SBP and DBP in the uncontrolled group, the age of the patients with uncontrolled blood pressure was higher than those with controlled blood pressure (63.67 ± 11.00 vs. 58.42 ± 10.07 years; p = 0.007). The number of DDIs and number of received antihypertensive medications were higher in the uncontrolled group; however, the differences did not reach a statistically significant level (for the number of DDIs, controlled vs. uncontrolled: 3.25 ± 4.19 vs. 4.86 ± 5.21, p = 0.067; for the number of received antihypertensive medications, controlled vs. uncontrolled: 2.13 ± 0.94 vs. 2.44 ± 0.97, p = 0.067). BMI (kg/m^2^), pulse rate/min, the total number of drugs, EQ5D index scores, and EQ scale scores did not differ between the controlled and uncontrolled groups. The differences between the controlled and uncontrolled groups are presented in Tables 1, 2.

**Table 1:** The differences in study parameters between the controlled and uncontrolled groups BMI, body mass index; SBP, systolic blood pressure; DBP, diastolic blood pressure; EQ-5D, EuroQol-5D; VAS, visual analog scale; DDIs, drug-drug interactions; *p < 0.05

	Controlled patients (n = 53)		Uncontrolled patients (n = 70)		
Parameter	Mean ± SD	Median (25^th^-75^th^ percentiles)	Mean ± SD	Median (25^th^-75^th^ percentiles)	p-value
Age (year)	58.42 ± 10.07	58 (51.50-63.50)	63.67 ± 11.00	65 (56-71)	0.007*
BMI (kg/m^2^)	31.85 ± 5.98	30.98 (26.62-35.34)	30.75 ± 4.99	30.19 (27.02-34.07)	0.649
SBP (mmHg)	127.04 ± 9.28	128 (121.50-134.50)	153.81 ± 12.24	149.50 (145-160)	<0.001*
DBP (mmHg)	75.70 ± 6.59	76 (72-81.50)	83.79 ± 10.56	85.50 (76.75-92)	<0.001*
Pulse rate/min	81.26 ± 10.88	79 (74.50-86.50)	81.54 ± 11.41	80 (73-90)	0.722
Number of antihypertensive drugs	2.13 ± 0.94	2 (1-3)	2.44 ± 0.97	3 (2-3)	0.067
Total number of drugs	4.53 ± 2.64	4 (2.5-6)	5.37 ± 2.93	5 (3-7.25)	0.111
EQ-5D index score	0.63 ± 0.24	0.62 (0.48-0.78)	0.67 ± 0.18	0.69 (0.60-0.78)	0.395
EQ-VAS score	72.17 ± 17.91	70 (60-80)	70.79 ± 18.53	70 (50-90)	0.442
Number of DDIs	3.25 ± 4.19	1 (0-6)	4.86 ± 5.21	3 (0-7.25)	0.067

**Table 2:** Gender distribution, presence of diabetes, and dyslipidemia in study groups

	Controlled patients (n = 53)	Uncontrolled patients (n = 70)	p-value
Gender (female/male)	35/18 (66% female)	47/23 (67% female)	0.898
Presence of diabetes	13 (25%)	25 (36%)	0.184
Presence of dyslipidemia	17 (32%)	29 (41%)	0.288

Correlations between study parameters

There was a significant linear correlation between age and SBP (r = 0.324, p < 0.001) and an inverse correlation between DBP and age (r = −0.208, p = 0.021). The number of DDIs showed significant correlations, positively with age (r = 0.303, p = 0.001), the total number of drugs (r = 0.784, p < 0.001), the number of antihypertensive drugs (r = 0.640, p < 0.001), and BMI (r = 0.321, p < 0.001) and inversely with EQ-5D index scores (r = −0.247, p = 0.006). On the other hand, the EQ-5D index score was inversely correlated with age (r = −0.199, p = 0.027), the number of total drugs (r = −0.230, p = 0.011), and BMI (r = −0.324, p < 0.001). Also, the EQ-5D VAS score was inversely correlated with age (r = −0.176, p = 0.051) and BMI (r = −0.343, p < 0.001). There were additional significant correlations between age and the total number of drugs (r = 0.431, p < 0.001), age and the number of antihypertensive drugs (r = 0.215, p = 0.017), the number of antihypertensive drugs and BMI (r = 0.239, p = 0.008), and the total number of drugs and BMI (r = 0.271, p = 0.002). As expected, there was also a strong correlation between the number of antihypertensive drugs and the total number of drugs (r = 0.614, p < 0.001). The correlations between study parameters are given in Table 3.

**Table 3:** The correlations between study parameters BMI, body mass index; SBP, systolic blood pressure; DBP, diastolic blood pressure; EQ-5D, EuroQol-5D; VAS, visual analog scale *p < 0.05, **p < 0.001

		SBP	DBP	Age	BMI	Number of antihypertensive drugs	Total number of drugs	Number of drug interactions	EQ index score	EQ scale
SBP	Correlation coefficient		0.421^**^	0.324^**^	-0.138	0.127	0.152	0.160	0.056	-0.019
	p-value		<0.001	<0.001	0.127	0.161	0.092	0.077	0.542	0.833
DBP	Correlation coefficient	0.421^**^		-0.208^*^	0.066	0.064	-0.040	0.012	0.066	-0.081
	p-value	<0.001		0.021	0.471	0.480	0.658	0.895	0.469	0.374
Age	Correlation coefficient	0.324^**^	-0.208^*^		-0.079	0.215^*^	0.431^**^	0.303^**^	-0.199^*^	-0.176
	p-value	<0.001	0.021		0.388	0.017	<0.001	0.001	0.027	0.051
BMI	Correlation coefficient	-0.138	0.066	-0.079		0.239^**^	0.271^**^	0.321^**^	-0.324^**^	-0.343^**^
	p-value	0.127	0.471	0.388		0.008	0.002	<0.001	<0.001	<0.001
Number of antihypertensive drugs	Correlation coefficient	0.127	0.064	0.215^*^	0.239^**^		0.614^**^	0.640^**^	-0.126	-0.104
	p-value	0.161	0.480	0.017	0.008		<0.001	<0.001	0.166	0.251
Total number of drugs	Correlation coefficient	0.152	-0.040	0.431^**^	0.271^**^	0.614^**^		0.784^**^	-0.230^*^	-0.138
	p-value	0.092	0.658	<0.001	0.002	<0.001		<0.001	0.011	0.127
Number of DDIs	Correlation coefficient	0.160	0.012	0.303^**^	0.321^**^	0.640^**^	0.784^**^		-0.247^*^	-0.152
	p-value	0.077	0.895	0.001	<0.001	<0.001	<0.001		0.006	0.094
EQ-5D index score	Correlation coefficient	0.056	0.066	-0.199^*^	-0.324^**^	-0.126	-0.230^*^	-0.247^*^		0.535^**^
	p-value	0.542	0.469	0.027	<0.001	0.166	0.011	0.006		<0.001
EQ-VAS score	Correlation coefficient	-0.019	-0.081	-0.176	-0.343^**^	-0.104	-0.138	-0.152	0.535^**^	
	p-value	0.833	0.374	0.051	<0.001	0.251	0.127	0.094	<0.001	

Evaluation of drug-drug interactions

At least one DDI was identified in 67.5% (83/123) of the study population. Of these, 21.7% (18/83) had only DDIs between antihypertensive drugs, and the vast majority were not remarkable in daily practice. In the present study, 511 DDIs were detected using the Medscape Drug Interaction Checker. Of these, 14 interactions in 12 patients were considered serious (use an alternative medication), 402 interactions in 82 patients were considered significant (monitor closely), and 95 interactions in 39 patients were considered minor. Additionally, among all identified 511 DDIs, 168 were between antihypertensive drugs. Hypertensive patients with diabetes mellitus had a higher number of significant DDIs (z = −3.778, p < 0.001), minor DDIs (z = −5.186, p < 0.001), and total number of DDIs (z = −4.493, p < 0.001) than hypertensive patients without diabetes mellitus. The number of serious DDIs did not differ between the patients with and without diabetes mellitus. Hypertensive patients with dyslipidemia had a higher number of significant (z = −3.343, p = 0.001), minor (z = −2.062, p = 0.039), and total number of DDIs (z = −3.564, p < 0.001) than the hypertensive patients without dyslipidemia. However, the number of serious DDIs did not differ between the patients with and without dyslipidemia. All serious DDIs are given in Table 4. Additionally, the 10 most common drug pairs that showed significant and minor interactions are presented in Tables 5, 6, respectively.

**Table 4:** Serious drug-drug interactions among prescribed drugs Severity, mechanism, and recommendations were given according to the Medscape Drug Interaction Checker Database. NSAIDs, non-steroidal anti-inflammatory drugs; ACE, angiotensin-converting enzyme; CYP3A4, cytochrome P450 3A4; CYP2C8, cytochrome P450 2C8; PD, pharmacodynamic; QTc, corrected QT; pH, potential of hydrogen

Drug pairs	Frequency	Mechanism of interaction	Recommendation
Aspirin-ramipril	4	Coadministration may result in a significant decrease in renal function. NSAIDs may diminish the antihypertensive effect of ACE inhibitors. The mechanism of these interactions is likely related to the ability of NSAIDs to reduce the synthesis of vasodilating renal prostaglandins.	Avoid or use an alternate drug.
Nifedipine-simvastatin	2	Nifedipine will increase the level or effect of simvastatin by affecting hepatic/intestinal enzyme CYP3A4 metabolism.	Avoid or use an alternate drug.
Amlodipine-simvastatin	1	Amlodipine increases levels of simvastatin.	Avoid or use an alternate drug. Benefits of combination therapy should be carefully weighed against the potential risks of combination. Potential for increased risk of myopathy/rhabdomyolysis. Limit simvastatin dose to no more than 20 mg/day when used concurrently.
Clopidogrel-repaglinide	1	Clopidogrel will increase the level or effect of repaglinide. Clopidogrel inhibits CYP2C8. Coadministration significantly increases repaglinide serum levels.	Avoid or use an alternate drug. If unable to avoid coadministration, decrease the initial repaglinide dose to 0.5 mg/meal and do not exceed 4 mg/day.
Diclofenac-quinapril	1	PD antagonism. Coadministration may result in a significant decrease in renal function. NSAIDs may diminish the antihypertensive effect of ACE inhibitors. The mechanism of these interactions is likely related to the ability of NSAIDs to reduce the synthesis of vasodilating renal prostaglandins.	Avoid or use an alternate drug.
Fluoxetine-indapamide	1	Fluoxetine and indapamide both increase QTc interval.	Avoid or use an alternate drug.
Lansoprazole-mesalamine	1	Lansoprazole decreases effects of mesalamine by increasing gastric pH. Applies only to the oral form of both agents. Applies only to sustained release dosage form.	Avoid or use an alternate drug.
Losartan-ramipril	1	Either increases the toxicity of the other by pharmacodynamic synergism. Dual blockade of the renin-angiotensin system increases risks of hypotension, hyperkalemia, and renal impairment.	Avoid or use an alternate drug.
Trifluoperazine-maprotiline	1	Trifluoperazine and maprotiline both increase QTc interval.	Avoid or use an alternate drug.
Verapamil-simvastatin	1	Verapamil will increase the level or effect of simvastatin by affecting hepatic/intestinal enzyme CYP3A4 metabolism. Potential for increased risk of myopathy/rhabdomyolysis.	Avoid or use an alternate drug. Do not exceed 10 mg of simvastatin daily when given concurrently.

**Table 5:** The 10 most common drug pairs that showed significant interactions Severity, mechanism, and recommendations were given according to the Medscape Drug Interaction Checker Database. NSAIDs, non-steroidal anti-inflammatory drugs; OATP1B1, organic anion transporting polypeptide 1B1

Drug pairs	Frequency	Mechanism of interaction	Recommendation
Valsartan-aspirin	35	Either increases the toxicity of the other by other. Aspirin decreases effects of valsartan by pharmacodynamic antagonism. Valsartan and aspirin both increase serum potassium.	Use caution/monitor. Both may result in renal function deterioration, particularly in elderly or volume-depleted individuals. Modify therapy/monitor closely. NSAIDs decrease the synthesis of vasodilating renal prostaglandins and thus affect fluid homeostasis and may diminish the antihypertensive effect.
Valsartan-hydrochlorothiazide	33	Valsartan increases and hydrochlorothiazide decreases serum potassium.	Effect of interaction is not clear; use caution. Use caution/monitor.
Atorvastatin-valsartan	27	Atorvastatin will increase the level or effect of valsartan by other. Valsartan increases toxicity of atorvastatin by other.	Use caution/monitor. The results from an in vitro study with human liver tissue indicate that valsartan is a substrate of the hepatic uptake transporter OATP1B1; coadministration with OATP1B1 inhibitors may increase valsartan systemic exposure. Use caution/monitor. Comment: OATP1B1 inhibitors may increase risk of myopathy.
Carvedilol-valsartan	19	Pharmacodynamic synergism. Valsartan and carvedilol both increase serum potassium.	Use caution/monitor. Risk of fetal compromise if given during pregnancy. Use caution/monitor
Hydrochlorothiazide-metoprolol	18	Either increases the toxicity of the other by other. Metoprolol increases and hydrochlorothiazide decreases serum potassium.	Modify therapy/monitor closely. It may cause idiosyncratic reactions, resulting in acute transient myopia and acute angle-closure glaucoma, which can lead to permanent vision loss. Effect of interaction is not clear; use caution. Use caution/monitor.
Valsartan-metoprolol	15	Pharmacodynamic synergism. Valsartan and metoprolol both increase serum potassium.	Use caution/monitor. Risk of fetal compromise if given during pregnancy. Use caution/monitor
Aspirin-hydrochlorothiazide	14	Aspirin increases and hydrochlorothiazide decreases serum potassium.	Effect of interaction is not clear; use caution. Use caution/monitor.
Amlodipine-metformin	12	Amlodipine decreases effects of metformin by pharmacodynamic antagonism.	Use caution/monitor. Patients should be closely observed for loss of blood glucose control; when drugs are withdrawn from a patient receiving metformin, the patient should be observed closely for hypoglycemia.
Losartan-aspirin	12	Either increases toxicity of the other by other. Aspirin decreases effects of losartan by pharmacodynamic antagonism. Losartan and aspirin both increase serum potassium.	Use caution/monitor. Comment: it may result in renal function deterioration, particularly in elderly or volume-depleted individuals. Modify therapy/monitor closely. NSAIDs decrease the synthesis of vasodilating renal prostaglandins and thus affect fluid homeostasis and may diminish the antihypertensive effect. Use caution/monitor.
Nebivolol-valsartan	12	Pharmacodynamic synergism. Both increase serum potassium	Use caution/monitor. Risk of fetal compromise if given during pregnancy. Use caution/monitor.

**Table 6:** The 10 most common drug pairs that showed minor interactions Severity, mechanism, and recommendations were given according to the Medscape Drug Interaction Checker Database. OATP1B1, organic anion transporting polypeptide 1B1

Drug pairs	Frequency	Mechanism of interaction	Recommendation
Hydrochlorothiazide-metformin	32	Hydrochlorothiazide will increase the level or effect of metformin by basic (cationic) drug competition for renal tubular clearance. Hydrochlorothiazide decreases effects of metformin by pharmacodynamic antagonism.	Minor/significance unknown. Minor/significance unknown. Thiazide dosage of >50 mg/day may increase blood glucose.
Hydrochlorothiazide-aspirin	14	Hydrochlorothiazide will increase the level or effect of aspirin by acidic (anionic) drug competition for renal tubular clearance.	Minor/significance unknown.
Hydrochlorothiazide-vildagliptin	4	Hydrochlorothiazide decreases effects of vildagliptin by pharmacodynamic antagonism.	Minor/significance unknown. Thiazide dosage of >50 mg/day may increase blood glucose.
Nifedipine-metformin	4	Nifedipine increases levels of metformin by enhancing GI absorption.	Applies only to oral form of both agents. Minor/significance unknown.
Valsartan-simvastatin	3	Valsartan increases toxicity of simvastatin by other.	Minor/significance unknown. Comment: OATP1B1 inhibitors may increase risk of myopathy.
Hydrochlorothiazide-verapamil	2	Hydrochlorothiazide will increase the level or effect of verapamil by basic (cationic) drug competition for renal tubular clearance.	Minor/significance unknown.
Hydrochlorothiazide-sitagliptin	2	Hydrochlorothiazide decreases effects of sitagliptin by pharmacodynamic antagonism.	Minor/significance unknown. Thiazide dosage of >50 mg/day may increase blood glucose.
Indapamide-metformin	2	Indapamide decreases effects of metformin by pharmacodynamic antagonism.	Minor/significance unknown. Thiazide dosage of >50 mg/day may increase blood glucose.
Verapamil-aspirin	2	Verapamil increases effects of aspirin by unknown mechanism.	Minor/significance unknown. Enhanced antiplatelet activity.
Famotidine-metformin	2	Famotidine increases levels of metformin by decreasing renal clearance.	Minor/significance unknown.

## Discussion

Hypertension is one of the most common chronic diseases and the most important preventable risk factor for cardiovascular diseases. According to the results of the TEKHARF study published in 2017, it was estimated that hypertension affected approximately 6 million male and 8 million female patients in Turkey [8].

The association of hypertension with increasing age is well known. The increased prevalence of the disease is also related to the increasing aging population [9]. The management and control of hypertension in the geriatric population need close monitoring and intensive follow-up. One of the main reasons for insufficient control of hypertension in older patients can be related to the comorbid conditions that need additional medications. It is a fact that polypharmacy can reduce compliance with medication, especially in the geriatric population [10]. In addition to the possible direct effects of comorbid conditions and related medications on blood pressure levels, polypharmacy may also lead to certain DDIs with antihypertensive drugs and decrease or increase the effect and/or adverse effects of antihypertensive treatment. In our study, it was demonstrated that the age of the patients with uncontrolled blood pressure was significantly higher than that of those with controlled blood pressure. Additionally, we found significant correlations between age and the total number of prescribed drugs, the number of antihypertensive drugs, drug interactions, and EQ-5D index scores. It can be concluded that age by itself is related to a higher incidence of uncontrolled hypertension and has an association with the need for multiple medications, thus increasing the risk of DDIs, which may contribute to a decrease in the quality of life of patients.

Our data were also consistent with the findings of a recent study that highlighted the association of increasing age and polypharmacy with DDIs in patients with hypertension [11]. In the present study, age also correlated positively with SBP and inversely with DBP. This finding may support previous knowledge that a wider pulse pressure level is an additional important risk factor for cardiovascular diseases in older patients [12]. On the other hand, the number of DDIs and the number of prescribed antihypertensive drugs tended to be higher in the uncontrolled group, though the difference did not reach a significant level, possibly due to the small sample size.

Obesity is an important public health challenge, and the increased prevalence in recent years carries a great risk for the development of hypertension and cardiovascular diseases [13]. In our study, the BMI score was positively correlated with the total number of prescribed drugs (r = 0.239, p = 0.008), the number of antihypertensive drugs (r = 0.271, p = 0.002), and DDIs (r = 0.321, p < 0.001). This finding may suggest that the patients with higher BMI scores have a higher number of comorbid conditions and use multiple drug treatments, thus leading to a drug interaction risk. The quality of life was also inversely correlated with BMI values, evidenced by both EQ-5D index scores (r = −0.324, p < 0.001) and EQ-5D VAS scores (r = −0.343, p < 0.001). These findings support that high BMI scores are one of the major risk factors contributing to the quality of life and management of antihypertensive treatment. Therefore, awareness should be increased globally, and patients should be informed to avoid possible complications of obesity and DDIs.

The most common comorbid conditions were diabetes mellitus and dyslipidemia among the study participants. The direct effect of both diseases on blood pressure levels could not be compared because the sample size and population of the study were not appropriate for this evaluation. The higher prevalence of hypertension among patients with diabetes and/or dyslipidemia [14,15] should be taken into consideration, and their effects on blood pressure levels should not be ignored. As an expected result, the presence of both conditions increased the risk of DDIs, including significant and minor interactions. However, the number of serious drug interactions did not significantly differ between the groups. The incidence of serious drug interaction was very low in the entire study population due to the small sample size and the data from a specialized clinical pharmacology polyclinic. Therefore, the risk of serious DDIs in subgroups should be evaluated in further studies surrounding larger populations.

One of the aims of the study was to identify the drug pairs that interact with each other and to determine the severity and mechanism of the DDIs in patients with hypertension. According to the Medscape Drug Interaction Checker Database, the possible recommendations were extracted and interpreted. In a previous study that aimed to evaluate the antihypertensive drug interactions for nine months after baseline, it was reported that approximately 75% of the patients had at least one interaction at baseline, and the number of interactions increased over time [16]. In our study, 67.4% (83/123) of the patients had at least one drug interaction that was compatible with the existing literature. The results of our study and previous knowledge support that DDIs should be taken into consideration for the successful management of antihypertensive treatment. Even in our relatively small study population, a remarkable number of 14 serious, 402 significant, and 95 minor interactions were detected. Only two of these 14 serious interactions did not involve antihypertensive medications. It is a fact that the vast majority of serious DDIs can be considered avoidable. The interaction between simvastatin and calcium channel blockers can be given as an example. In this case, replacement of treatments with appropriate alternatives, such as the use of a different statin not metabolized through the CYP3A4 pathway or changing the antihypertensive treatment regimen, can reduce the risk of serious complications such as rhabdomyolysis.

The vast majority of the DDIs fell under the category of significant interactions (monitor closely). Most common five drug pairs that showed a significant interaction were valsartan-aspirin, valsartan-hydrochlorothiazide, atorvastatin-valsartan, carvedilol-valsartan, and hydrochlorothiazide-metoprolol. Pharmacodynamic interactions between antihypertensive drugs were not remarkable for daily practice because the synergy between the blood pressure lowering effects of antihypertensives is a natural outcome, and it does not indicate that these drugs should not be used concomitantly. It is well-known that, as mentioned before, guideline recommendations for patients with markedly high initial blood pressure levels are to initiate treatment with an appropriate combination of antihypertensive drugs. However, other significant interactions require close monitoring, such as serum potassium levels with concomitant use of carvedilol and valsartan or valsartan-hydrochlorothiazide, and if possible, stopping or replacing the concomitant treatment, or modifying treatment with concomitant use of hydrochlorothiazide and metoprolol to protect patients from eye/vision-related complications.

Our results indicated that the interactions between aspirin and renin-angiotensin-aldosterone system (RAAS) blockers required special emphasis because both are widely used in patients with cardiovascular disease, and their interactions may be overlooked in daily practice. Even in our relatively small sample size, there were four serious, 61 significant, and two minor interactions between aspirin and other cardiovascular drugs. The serious interaction between aspirin and ramipril, an angiotensin-converting enzyme (ACE) inhibitor, is based on the possible risk of a decrease in renal function, and additional interaction is caused by non-steroidal anti-inflammatory drugs (NSAIDs) inhibiting vasodilating renal prostaglandins, thus disrupting the blood pressure lowering effects of ACE inhibitors. The other significant (monitored closely) interaction between these drugs is based on impaired renal functions, especially in the elderly and patients with volume depletion, according to the Medscape Drug Interaction Database.

Although there are no serious interactions between aspirin and valsartan (the most commonly used angiotensin-receptor blocker [ARB] in the study), there are significant interactions that require close monitoring. The mechanism of significant interactions between aspirin and valsartan is very similar to the interaction of ramipril with aspirin; however, the severity level is not the same. The mechanism and severity of the DDIs of aspirin with other ACE inhibitors (including perindopril, lisinopril, and trandolapril) and ARBs (including candesartan, losartan, and telmisartan) were the same. With the high risk of drug interaction of aspirin with antihypertensive drugs, the patients should first be evaluated for an aspirin indication. The proven benefit of aspirin in the secondary prevention of cardiovascular diseases is well established; however, the effect of aspirin in the primary prevention of cardiovascular diseases is not clear due to a poorly defined balance of benefit and bleeding risk. Accordingly, the recommendations and guidelines on the use of aspirin in primary prevention are very restricted [17]. Therefore, the concomitant use of aspirin in the treatment of patients with hypertension should be carefully evaluated because both RAAS blockers and aspirin are cornerstone medications in the treatment of several atherosclerotic cardiovascular diseases.

In other cardiovascular conditions, such as heart failure, however, the concomitant use of aspirin and ACE inhibitors is debatable. It was stated in a previous publication that the risk of drug interactions between aspirin and ACE inhibitors could be associated with the severity of heart failure; therefore, avoiding the use of aspirin and other NSAIDs, and if needed, the use of other antiplatelet agents not disrupting the prostaglandin synthesis, such as clopidogrel, might be considered in patients with severe heart failure [18]. In general, we recommend that if aspirin is clearly indicated and needs to be used with a RAAS blocker, use of a low-dose aspirin range of 75-100 mg (in line with guidelines’ recommendation [17]), selection of ARBs without serious interaction with aspirin, and maintaining close monitoring of renal functions may be a better approach until new evidence comes into view.

The results of our study demonstrated that DDIs were related to decreased quality of life. Both the EQ-5D index and VAS scores tended to demonstrate an inverse correlation with the number of DDIs, but only the correlation of the EQ-5D index score (r = −0.247, p = 0.006) reached significance. However, this may be enough for a clinical evaluation because VAS scores are a more subjective assessment based only on patient consideration. In this sense, this finding revealed that the successful management of DDIs has the potential to improve the quality of life in patients with hypertension.

Limitations

The main limitation was the relatively small sample size of this single-center study. However, even in our relatively small study population, the remarkable number of DDIs detected showed the importance of the management of DDIs in patients with hypertension. On the other hand, this study has a cross-sectional design; therefore, the results provide only a snapshot of the current situation. It should be kept in mind that the present study demonstrates the correlations but not causation between study variables due to the study design and statistical analysis. According to the detected DDIs, appropriate alteration in treatment plans and their effects on blood pressure levels and quality of life should be evaluated further in future studies.

## Conclusions

The results of the present study are important in highlighting the association of DDIs in the management of antihypertensive treatment and the quality of life of patients. Additionally, the increased need for multiple medications, increased risk of DDIs, and decreased quality of life were demonstrated in patients with older age and higher BMI scores. Among the detected DDIs, the interactions between aspirin and RAAS blockers required special emphasis. The need for aspirin use should be thoroughly considered in line with guideline recommendations. Regarding current knowledge, if aspirin is indicated and needs to be used with a RAAS blocker, the better approach may be to select an ARB without serious interaction with aspirin and maintain close monitoring of renal functions.
